# A high-throughput neutralizing assay for antibodies and sera evaluation against Epstein-Barr virus

**DOI:** 10.1186/s12985-022-01911-1

**Published:** 2022-11-23

**Authors:** Ling Zhong, Claude Krummenacher, Wanlin Zhang, Junping Hong, Qisheng Feng, Qinjian Zhao, Yixin Chen, Mu-Sheng Zeng, Yi-Xin Zeng, Miao Xu, Xiao Zhang

**Affiliations:** 1 State Key Laboratory of Oncology in South China, Collaborative Innovation Center for Cancer Medicine, Department of Experimental Research, Sun Yat-Sen University Cancer Center, Sun Yat-Sen University, Guangzhou, Guangdong People’s Republic of China; 2 College of Pharmacy, Chongqing Medical University, Chongqing, People’s Republic of China; 3 State Key Laboratory of Molecular Vaccinology and Molecular Diagnostics, National Institute of Diagnostics and Vaccine Development in Infectious Diseases, School of Public Health, Xiamen University, Xiamen, Fujian People’s Republic of China; 4 Department of Biological and Biomedical Sciences, Rowan University, Glassboro, NJ USA

**Keywords:** Epstein–Barr virus, High–throughput assay, Neutralizing antibodies, Human sera, Monkey sera, Anti–gp350 antibody 72A1, Anti–gHgL antibody CL59

## Abstract

**Background:**

Epstein-Barr virus (EBV) is a wide-spread human herpesvirus that is highly associated with infectious mononucleosis and several malignancies. Evaluation of EBV neutralizing antibody titers is important for serological studies, vaccine development and monoclonal antibody screening. The traditional method based on antibody inhibition of EBV transformation of B cells is very time-consuming. A more practical flow cytometry-based (FCM) approach to evaluate neutralizing titers is not amenable to achieving high-throughput evaluation of large-scale samples. A high-throughput approach is urgently needed.

**Results:**

Here, we present a rapid and high-throughput method based on high content imaging system (HCIS) analysis. EBV titers determined by the HCIS-based assay were similar to those obtained by the FCM-based assay. Neutralizing titers of sera and monoclonal antibodies measured by the HCIS-based assay strongly correlated with titers measured by the FCM-based assay. HCIS assays showed a strong correlation between B cell infection neutralizing titers and the anti-gp350 IgG titers in healthy EBV carriers and monkey sera. Finally, anti-gHgL IgG titers from sera of healthy EBV carriers significantly correlated with epithelial cell infection neutralizing titers.

**Conclusions:**

This HCIS-based assay is a high-throughput assay to determine viral titers and evaluate neutralizing potentials of sera and monoclonal antibodies. This HCIS-based assay will aid the development of vaccines and therapeutic monoclonal antibody against EBV.

**Supplementary Information:**

The online version contains supplementary material available at 10.1186/s12985-022-01911-1.

## Background

Epstein-Barr virus (EBV) is the leading aetiological agent of infectious mononucleosis and several malignancies including nasopharyngeal carcinoma (NPC), gastric carcinoma (GC), Burkitt lymphoma (BL), Hodgkin lymphoma (HL) and natural killer (NK) /T cell lymphoma [1]. EBV is also associated with autoimmune diseases such as systemic lupus erythematosus, Sjögren’s syndrome and rheumatoid arthritis [2]. Recently, a longitudinal study reported that the risk of multiple sclerosis (MS) increased 32-fold after EBV infection [3]. EBV causes heavy global public health burdens with 113,205, 105,554, 40,109 and 6,318 new cases per year of GC, NPC, HL and BL worldwide, respectively [4]. However, no vaccines against EBV infection or therapeutic agents for EBV-linked diseases are available.

During its infectious cycle, EBV exhibits two distinct tropisms toward epithelial cells and B cells. Virions derived from epithelial cells tend to infect B cells while virions produced by B cells more effectively infect epithelial cells [5]. These tropisms depend on the viral surface density of different glycoprotein complexes, gHgLgp42 and gHgL. During virus entry, additional glycoproteins, are involved in attachment to target cells (gp350 and BMRF2) and execution of membrane fusion (gB). The most abundant glycoprotein on the viral surface is gp350, which interacts with complement receptor 2 (CR2) [6] or CR1 [7] to initiate the infection of B cells. Following attachment, gHgLgp42 binds to human leukocyte antigens class II (HLA-II) and further triggers gB conformational changes to finish fusion [8]. Epithelial cell infection is initiated by BMRF2 binding to cellular integrins [9]. This is followed by gHgL binding to ephrin receptor A2 and activation of gB to execute membrane fusion [8, 10, 11].

Many serological studies have attempted to correlate antibodies elicited by EBV with infection status or disease outcomes [12–16]. Three parameters are commonly used to distinguish acute infection from past infection: viral capsid antigen (VCA)-IgG, VCA-IgM and EBV nuclear antigen 1 (EBNA1) -IgG [12]. The serological characteristics of healthy EBV carriers are VCA-IgM (−), VCA-IgG ( +), EBNA1-IgG ( +) and EBNA2-IgG (weak or -) [13]. Furthermore, levels of antibodies against various EBV proteins are predictive markers for the risk of developing NPC, GC, HL, BL and NK/T lymphoma [14, 15]. Another study revealed that high titers of antibodies targeting glycoproteins were detected in both NPC patients and healthy carriers, and sera from each group have similar neutralizing abilities [16]. Anti-gp350 antibodies are the major contributors to B cell neutralization, while anti-gHgL antibodies play an important role in epithelial cell neutralization [17].

Many prophylactic vaccine formulations against EBV infection have been studied since the 1980s. gp350 has been considered an ideal candidate for the development of prophylactic vaccines to prevent the initial EBV infection. Various gp350-based vaccine modalities including soluble recombinant proteins (multimeric and monomeric), viral vectors, nucleic acids, virus-like particles and nanoparticles were developed and evaluated in animal models [18]. Besides, clinical trials have been launched to evaluate gp350-based vaccines, including a recombinant vaccinia virus (Tien Tan strain) expressing gp350 [19], gp350 adjuvanted with alum or AS04 [20–22] and ferritin nanoparticles displaying gp350 (NCT0464514). Recently, a phase I clinical trial for an mRNA-based vaccine consisting of four mRNAs encoding gH, gL, gp42 and gp220 has also been initiated (mRNA-1189; NCT05164094).

EBV infection is a complicated process and humoral immune responses are important for EBV primary infection control. Neutralizing monoclonal antibodies are potential therapeutic agents and useful guides to improve vaccine design. To date, various neutralizing monoclonal antibodies targeting EBV envelope glycoproteins have been reported, including 72A1 (gp350) [23], AMMO1 (gHgL) [24], 6H2 (gHgL) [25], 1D8 (gHgL) [26], CL40 (gHgL) [27], CL59 (gHgL) [27], E1D1 (gL) [28], F-2–1 (gp42) [29], AMMO5 (gB) [24],3A3 (gB) [30], 3A5 (gB) [30], 8A9 (gB) [31] and 8C12 (gB) [31].

Determination of neutralizing titers is considered the critical index for serological studies and vaccine-induced humoral responses and is essential for monoclonal antibody screening. However, available approaches including inhibition of human B cell transformation, immunofluorescence-based assay, competition enzyme-linked immunosorbent assay (ELISA) and flow cytometry-based (FCM) based assay to determine neutralizing titers are time-consuming and unsuitable for testing large-scale clinical samples in high-throughput settings as discussed below [32–35]. A classical method to measure neutralizing titers is based on inhibition of human B cell transformation, which requires a 6–8 week detection period [32]. An immunofluorescence-based assay to detect EBV-positive stained Raji cells was developed but this early approach was limited by manual counting [33]. Alternatively, a competition ELISA using the neutralizing monoclonal antibody 72A1 provides a surrogate approach to detect the presence of neutralizing antibodies, but this assay does not determine actual titers [34]. Furthermore, an FCM neutralization assay utilizing B cells infected by EBV-GFP (green fluorescence protein) was developed, which is limited by the relatively low throughput at data collection and analysis [35]. Recently, a higher-throughput fluorescent imaging assay (FIA) using Akata-EBV-GFP to infect SVK-CR2 cells (an epithelial cell line overexpressing CR2) was reported, but it may not truly reflect the natural infection process [36, 37].

High content imaging system (HCIS) uses a high-throughput live cell imaging format and applies automated microscopy, fluorescent detection and multiparameter algorithms. HCIS has been used to visualize and quantify the interaction of therapeutics in cell populations [38]. Considering the high-throughput potential of image capture and analysis of HCIS, we developed a rapid and high-throughput method based on HCIS to determine neutralizing titers in B cells and epithelial cells. We validated this method in EBV infection of epithelial cell models (HNE1 epithelial cells infected with Akata-EBV-GFP virus) and B cell models (Akata B cells infected with CNE2-EBV-GFP virus). We compared the infection titers of CNE2-EBV-GFP and neutralizing titers of monoclonal antibodies determined by HCIS-based assays and FCM-based assays. A strong correlation was observed between CNE2-EBV-GFP viral titers defined by HCIS-based assay and FCM-based assay. The half maximal neutralizing concentration (NC_50_) of monoclonal antibody 72A1 or CL55 was also similar in both assays. We evaluated the neutralizing titers of sera from healthy EBV carriers and sera from monkeys infected with rhesus lymphocryptovirus (rhLCV), a simian homolog of EBV [39, 40]. Neutralizing titers in sera of healthy EBV carriers and infected monkeys determined by this HCIS-based assay in B cells and epithelial cells correlated highly with titers measured by the FCM-based assay. Finally, B cell neutralizing titers correlated with anti-gp350 IgG titers while anti-gHgL IgG titers correlated with epithelial cell neutralizing titers. This HCIS assay is a practical test with high-throughput potential, which will aid and facilitate further development of prophylactic vaccines and therapeutic treatments against EBV.

## Methods

### Human specimens

Sera were collected from age 40 to 60 EBV positive healthy carriers (VCA-IgM (−), VCA-IgG ( +), EBNA1-IgG ( +) and EBNA2-IgG (weak or -)) and their gender was documented by the investigators. This study was approved by the Institutional Ethics Committee of the Sun Yat-sen University Cancer Center, Guangdong, China. Written informed consent was obtained from all participants.

### Cells lines

All cell lines were cultured at 37 °C in humidified air containing 5% CO_2_. Akata cells (EBV negative, B cells) and HNE1 cells (EBV negative, epithelial cells) [41] were cultured in RPMI 1640 (Invitrogen) with 10% fetal bovine serum (FBS; Invitrogen), and antibiotics (penicillin, 100 U/ml; streptomycin, 100 μg/ml; Invitrogen). CNE2-EBV cells (epithelial cells) [42] and Akata-EBV cells (B cells) [43], were propagated in RPMI 1640 (Invitrogen) with 10% FBS (Invitrogen) and antibiotics (penicillin, 100 U/ml; streptomycin, 100 μg/ml; Invitrogen), and maintained under G418 selection (700 μg/ml; MP Biomedicals).

### Virus production

CNE2-EBV cells carrying the Akata-EBV-GFP genome were induced by 20 ng/ml 12-O-tetradecanoylphobol 13-acetate (TPA; Beyotime) and 2.5 mM sodium butyrate (NaB; Sigma Aldrich) for 12 h. After 72 h in culture, the supernatant was collected, centrifuged and then filtrated through a 0.45 μm filter to remove cell debris. The resulting virus, named CNE2-EBV-GFP, was concentrated 100 × by centrifugation at 50,000 g for 2.5 h and re-suspended by RPMI 1640 without FBS. The CNE2-EBV-GFP virions were stored at − 80 °C. A NIKON Eclipse Ti2-U microscope was used to capture images of non-induced CNE2-EBV cells as well as induced cells at 72 h post induction.

Akata-EBV cells carrying Akata-EBV-GFP were resuspended in RPMI 1640 without FBS and induced by 0.8% (v/v) goat anti-human IgG (Tianfun Xinqu Zhenglong Biochem. Lab). The medium was changed after 6 h induction. The Akata-EBV-GFP virus collection procedures and storage were the same as those used for the CNE2-EBV-GFP virus. A NIKON Eclipse Ti2-U microscope was used to capture images of non-induced Akata-EBV cells as well as induced cells at 72 h post induction.

### Transmission electron microscopy

EBV virions were observed by negative staining electron microscopy. Briefly, viral samples were applied to 200-mesh carbon-coated copper grids for 5 min. The excess solution was removed, grids were washed twice with double distilled water and immediately stained for 30 s with freshly filtered 1.6% phosphotungstic acid (pH 6.5). Grids were examined using an FEI Tecnai T12 TEM (FEI, USA) at an accelerating voltage of 120 kV and photographed at a magnification of 150,000 and 250,000 fold.

### CNE2-EBV-GFP virus titers definition by FCM

1 × 10^4^ Akata cells were seeded in each well of a 96-well plate in 180 μl RPMI 1640 with 10% FBS and incubated with 20 μl of twofold serially diluted CNE2-EBV-GFP virus at 37 °C in a 5% CO_2_ humidified atmosphere. After 48 h incubation, cells were collected by centrifugation at 500 g for 5 min and washed once with PBS. Cells were resuspended in PBS without fixation for observation. The infection efficiency (percentage of GFP-positive cells) was determined using a CytoFLEX S (Beckman Coulter) and analyzed using FlowJo software X 10.0.7 (Tree Star). Half maximal infection dilution fold (ID_50_) was determined by GraphPad Prism 8.0.

### CNE2-EBV-GFP virus titers definition by HCIS

1 × 10^4^ Akata cells were seeded in a 96-well plate in 180 μl RPMI 1640 with 10% FBS and incubated with 20 μl twofold serially diluted CNE2-EBV-GFP virus at 37 °C in a 5% CO_2_ humidified atmosphere. After 48 h incubation, the plate was shaken to disperse the cells and let them be evenly distributed in the well. Images were captured and the total GFP positive spots of each well were calculated using the Operetta CLS high content imaging system (PerkinElmer).

### Neutralizing titers evaluated by FCM

For B cell neutralization, 20 μl tenfold serially diluted monoclonal antibody 72A1 (starting from 100 μg/ml) or fivefold serially diluted sera from healthy EBV carriers or monkey (starting from 1:10) were mixed and incubated with 20 μl CNE2-EBV-GFP (a dose sufficient to infect 20% of cells) for 2 h at 37 °C in a 5% CO_2_ humidified atmosphere. The mixture was added to 1 × 10^4^ Akata cells and incubated for 48 h. Uninfected cells were used as negative controls and cells incubated with CNE2-EBV-GFP in the absence of antibody or sera were used as positive controls. The infected cells were counted using a CytoFLEX S (Beckman Coulter) and analyzed using FlowJo software X 10.0.7 (Tree Star). The neutralizing activity of each sample was calculated as (%GFP positive cells of positive control–%GFP positive cells of samples with antibody or sera) × 100/ %GFP positive cells of positive control. Half maximal neutralizing concentrations (NC_50_) for monoclonal antibody or half maximal neutralizing dilution folds (ND_50_) for sera were determined by GraphPad Prism 8.0.

For epithelial cell neutralization, 20 μl twofold serially diluted sera from healthy EBV carriers (starting from 1:10) were mixed and incubated with 20 μl Akata-EBV-GFP for 2 h at 37 °C in a 5% CO_2_ humidified atmosphere. The mixture was added to 0.4 × 10^4^ HNE1 cells and the medium was changed after 3 h. The following steps of data collection, analysis and calculation were the same as for the B cell neutralization.

### Neutralizing titers evaluated by HCIS

For the B cell neutralization model, 20 μl tenfold serially diluted monoclonal antibody 72A1 (starting from 100 μg/ml) or fivefold serially diluted sera from healthy EBV carriers or monkey (starting from 1:10) were mixed and incubated with 20 μl CNE2-EBV-GFP virus for 2 h at 37 °C in a 5% CO_2_ humidified atmosphere. The mixture was added to 1 × 10^4^ Akata cells and incubated for 48 h. Uninfected cells were used as negative controls and cells incubated with EBV in the absence of antibody or sera were used as positive controls. After 48 h incubation, the plate was shaken to disperse the cells to obtain even distribution in the wells. Images were captured and GFP positive spots were counted by Operetta CLS high content imaging system (PerkinElmer). The neutralizing rate of each sample was calculated as (number of total GFP positive spots of positive control–number of total GFP positive spots of samples with antibody or sera) × 100/ number of total GFP positive spots of positive control. Half maximal neutralizing concentrations (NC_50_) for monoclonal antibody or half maximal neutralizing dilution folds (ND_50_) for sera were determined by GraphPad Prism 8.0.

For the epithelial cell neutralizing model, 20 μl twofold serial diluted healthy EBV carriers sera (starting from 1:10) were mixed and incubated with 20 μl Akata-EBV-GFP for 2 h at 37 °C in a 5% CO_2_ humidified atmosphere. The mixture was added to 0.4 × 10^4^ HNE1 cells and the medium was changed after 3 h. The following steps of data collection, analysis and calculation were the same as for the B cell neutralization model.

### Enzyme-linked immunosorbent assay (ELISA)

Wells of 96-well ELISA plates (Corning) were coated with 100 ng/well gp350 or gHgL in PBS by incubation at 37 °C for 2 h. After washing with TBST (Tris Buffered Saline with Tween 20), blocking buffer (PBS containing 0.5% casein, 2% gelatin and 0.1% ProClin 300, pH 7.4) was used to block plates for 2 h at 37 °C. Five-fold serially diluted sera from monkeys or healthy EBV carriers (starting from 1:100) were added to each well, incubated for 1 h at 37 °C and then washed 5 times with TBST. Goat anti-human antibody conjugated with HRP (Promega) was added (1:5000 dilution) and incubated for 30 min at 37 °C. The colorimetric reaction was developed using the EL-TMB kit (Sangon Biotech). Absorbance was measured at 450 nm and 630 nm using a microplate reader (Molecular Devices).

### Statistics

The Spearman correlation coefficient was used to evaluate the correlation between the results of different assays.

## Results

### Production of CNE2-EBV-GFP virus

**Fig. 1:**
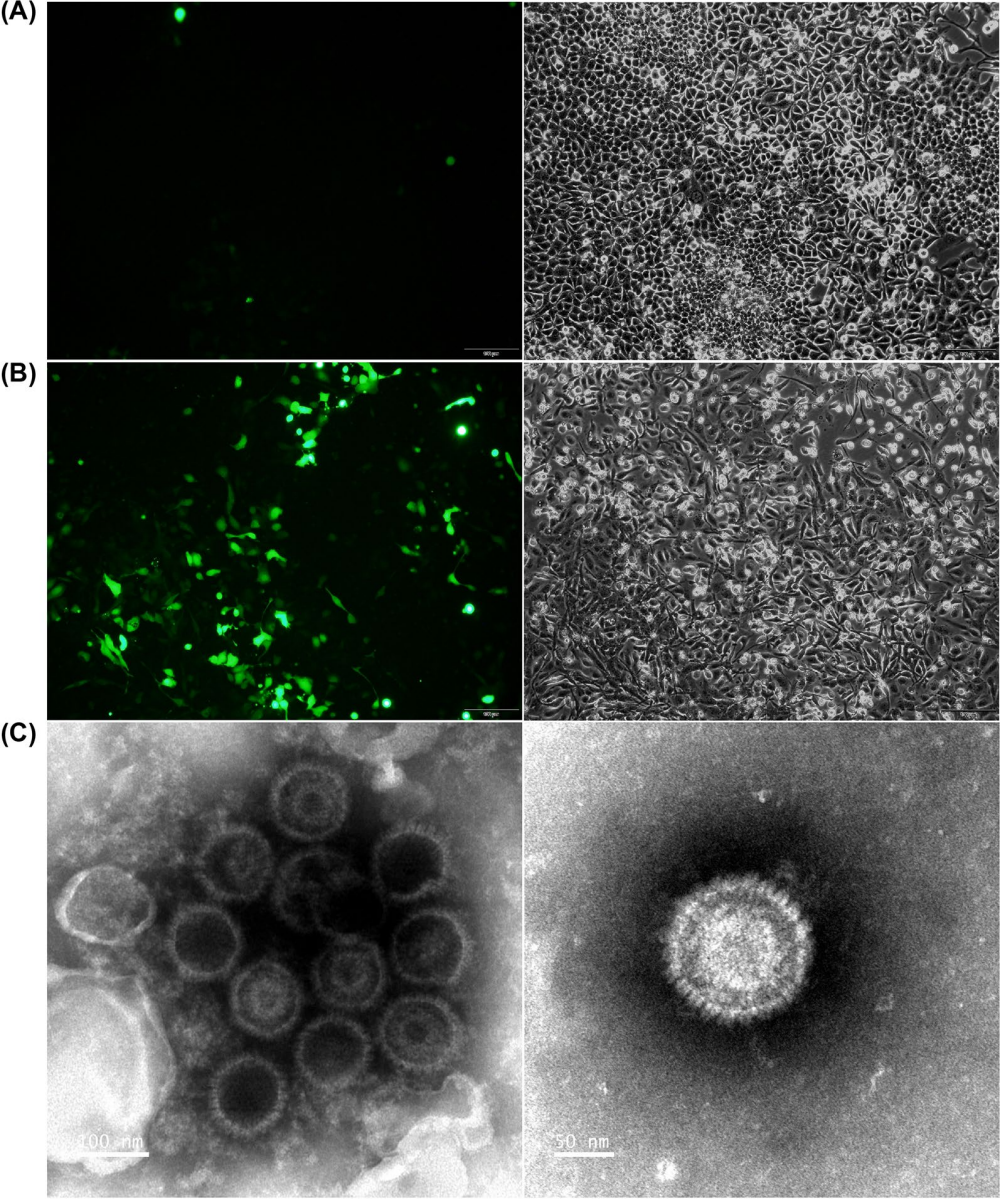
CNE2-EBV-GFP virus production and TEM detection. **A** Images of CNE2-EBV cells carrying Akata-EBV-GFP before induction. Few GFP spots were observed indicating that most viruses remained in a latent state. **B** Images of CNE2-EBV cells carrying Akata-EBV-GFP 72 h after induction. The majority of cells displayed GFP expression, indicating that EBV was induced to switch to a lytic phase of replication. For HCIS quantification, each GFP expressing cell is counted as a positive spot. (A-B). Images of cells with the same field of view captured at FITC channel (left panel) and bright-field channel (right panel). **C** TEM images of CNE2-EBV-GFP virus. Capsids are visible, but the viral envelope is not observed because sample processing for TEM observation disrupted this membrane structure. The magnification of the left channel is 150,000 x (scale bar: 100 nm) and that of the right channel is 250,000 × (scale bar: 50 nm)

CNE2-EBV cells carrying Akata-EBV-GFP were induced by TPA and NaB for 12 h. GFP fluorescence was readily observed 72 h after induction compared to non-induced cells (Fig. 1A and B). The increased number of GFP-expressing cells indicated that EBV switched to a lytic phase and was actively replicating. To confirm viral production, CNE2-EBV-GFP virions were collected from the culture medium and concentrated 100 × by centrifugation. The concentrated CNE2-EBV-GFP virions were visualized by transmission electron microscopy (Fig. 1C). Viral capsids are visible, but the viral envelope is not observed because sample processing for TEM observation disrupted this membrane structure.

### CNE2-EBV-GFP titers defined by FCM and HCIS

**Fig. 2:**
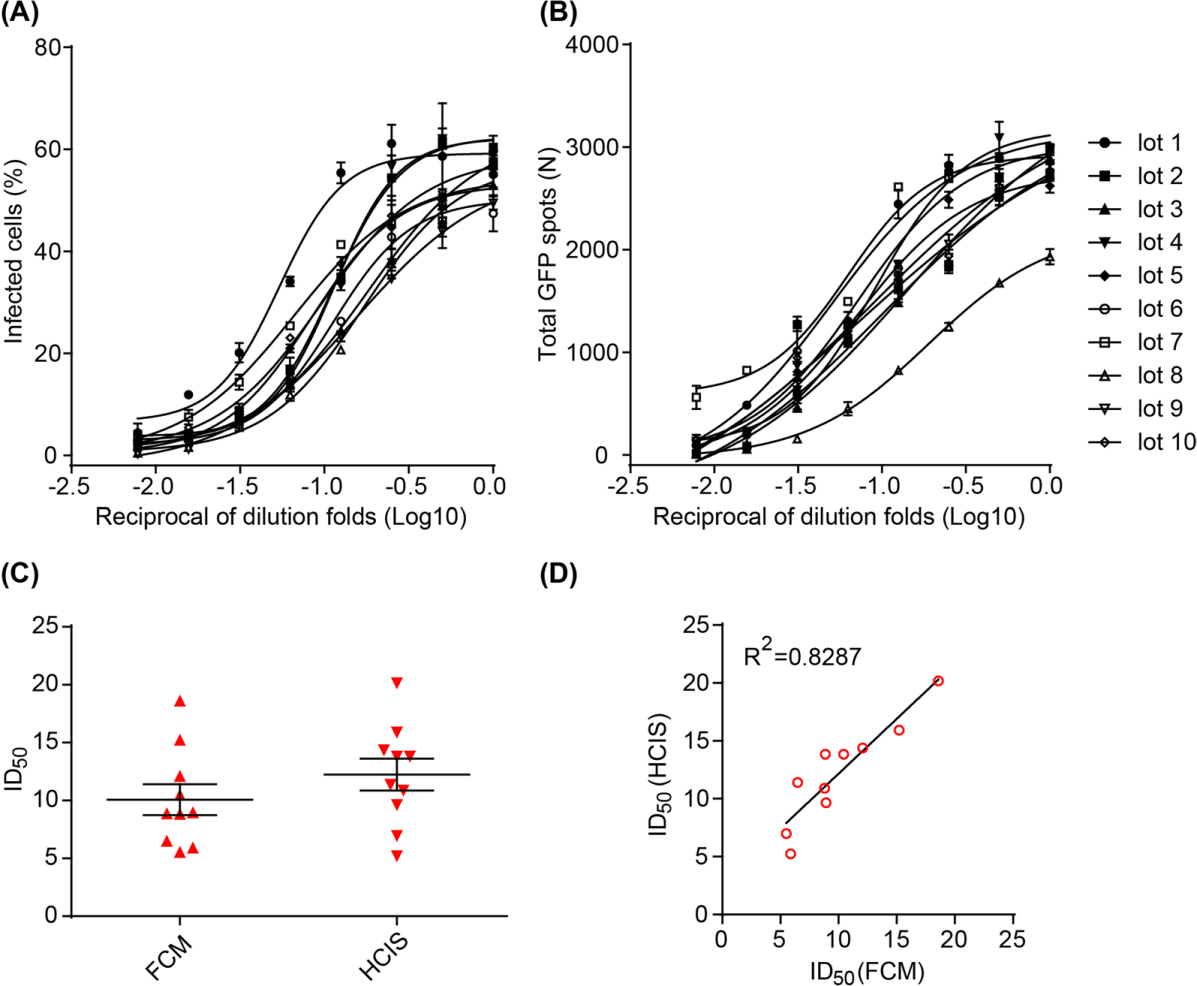
Infection titers of 10 lots of CNE2-EBV-GFP virus on Akata cells determined by FCM and HCIS. **A** Infection titers of 10 lots of CNE2-EBV-GFP virus determined by FCM. Data are shown as mean ± SEM (n = 2). Curves were fit using four parameters nonlinear regression. **B** Infection titers of 10 lots of CNE2-EBV-GFP virus determined by HCIS. Data are shown as mean ± SEM (n = 2). Curves were fit using four parameters nonlinear regression. One spot represents one cell and GFP positive spots correspond to infected cells. **C** Half maximal infection dilution fold (ID_50_) of 10 lots of virus defined by FCM and HCIS, respectively. Horizontal bars indicate mean ± SEM. **D** Correlation of ID_50_ values defined between FCM and HCIS for each of the 10 lots of CNE2-EBV-GFP

To determine whether the HCIS-based assay can be applied to measure EBV titers, we compared the titers of ten different lots of CNE2-EBV-GFP obtained by FCM (Fig. 2A) and HCIS (Fig. 2B). To quantify the HCIS assay, each cell is defined as a single spot and the infected cells were counted as GFP positive spots. CNE2-EBV-GFP virus was produced from an epithelial cell line, so that it does more efficiently infect B cells. As a target to evaluate CNE2-EBV-GFP titers, we utilized EBV negative B cell line, Akata. Serially diluted CNE2-EBV-GFP viruses were incubated with 1 × 10^4^ Akata cells and the infection ratios were determined 48 h after incubation. The infection ratio of FCM-based assay is shown as the percentage of GFP positive infected cells (Additional file 1: Fig. S1). For the HCIS-based assay, the infection was measured as the total number of GFP positive spots (Additional file 1: Fig. S1). The ID_50_ value was determined using four-parameter nonlinear regression. ID_50_ values determined by either FCM or HCIS ranged from 5 to 25 (Fig. 2C). Virus titers determined by HCIS or FCM showed a high degree of correlation (R^2^ = 0.8287; Fig. 2D), indicating that both methods are equally reliable. Thus, the HCIS assay provides a valuable GFP-based approach to determine EBV titers in B cells that is amenable to high-throughput analysis.

### Monoclonal antibody 72A1 neutralizing titers evaluated by FCM and HCIS

**Fig. 3:**
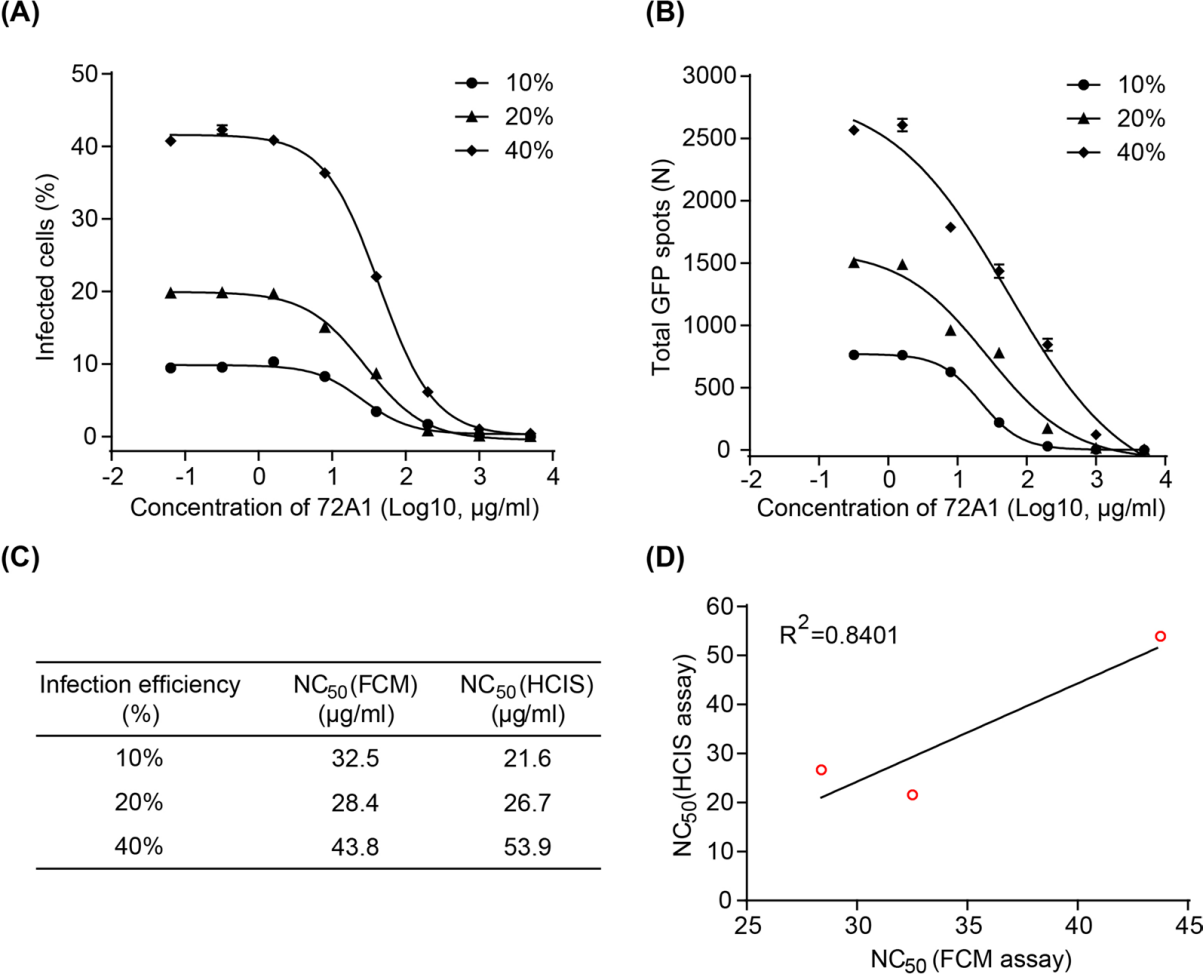
Neutralizing ability of monoclonal antibody 72A1 against CNE2-EBV-GFP infection of B cells determined by FCM and HCIS. **A** Neutralizing ability of 72A1 determined by FCM using doses of CNE2-EBV-GFP with different levels of infection efficiency (10%, 20% and 40%). Data are shown as mean ± SEM (n = 2). Curves were fit using four parameters nonlinear regression. **B** Neutralizing ability of 72A1 determined by HCIS using doses of CNE2-EBV-GFP with different levels of infection efficiency (10%, 20% and 40%). Data was shown as mean ± SEM (n = 2). Curves were fit using four parameters nonlinear regression. **C** Half maximal neutralizing concentration (NC_50_) of 72A1 determined by FCM and HCIS, for each dose of virus. **D** Correlation of NC_50_ values between FCM and HCIS assays

Monoclonal antibody 72A1 is a gp350-specific neutralizing antibody that potently blocks EBV infection of B cells by interfering with gp350 binding to its receptor CR2 [44]. To investigate whether the HCIS assay can be applied to assess neutralizing titers of EBV-specific monoclonal antibodies, we determined NC_50_ values (half neutralizing concentration) for 72A1 using different infectious doses of CNE2-EBV-GFP leading to infection of 10%, 20% or 40% of Akata cells. Virus inoculum was incubated with serially diluted 72A1 for 2 h before addition to cells. After 48 h incubation, the neutralizing ability of 72A1 was determined by FCM. The number of GFP-positive cells in the presence of antibody was compared to the positive control (no antibody) and neutralization curves were fit using four parameters nonlinear regression (Fig. 3A). In parallel, the 72A1 neutralizing activity was assessed by HCIS. The total number of GFP positive spots was determined and compared with the positive control (no antibody) and neutralization curves were fit using four parameters nonlinear regression (Fig. 3B) to determine NC_50_ values (Fig. 3C).

Monoclonal antibody 72A1 potently neutralized CNE2-EBV-GFP infection of Akata cells over a range of 10%, 20% and 40% infection efficiency. Importantly, NC_50_ values obtained by FCM and HCIS were similar (Fig. 3C). Overall, NC_50_ values obtained by HCIS and FCM showed a strong correlation (R^2^ = 0.8401; Fig. 3D). Hence, the HCIS-based assay can be applied to accurately determine the neutralizing efficiency of EBV-specific monoclonal antibodies. Compared to FCM, the HCIS approach will significantly reduce the workload of monoclonal antibody screening.

### Neutralizing titers of sera determined by FCM and HCIS in B cells

**Fig. 4:**
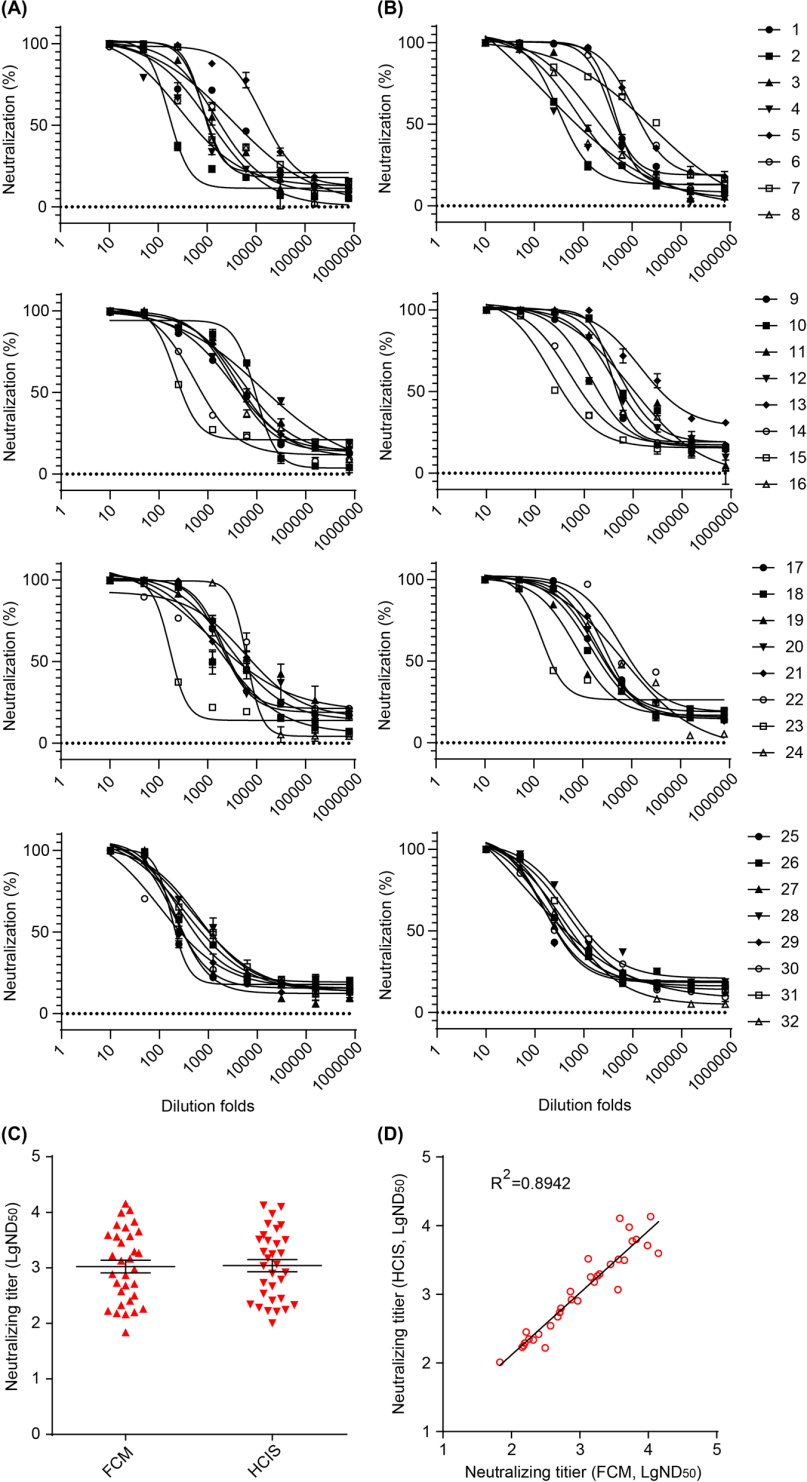
Neutralizing titers of 32 human sera CNE2-EBV-GFP virus infection of B cells determined by FCM and HCIS. **A** Neutralizing titers of 32 human sera determined by FCM. Data are shown as mean ± SEM (n = 2). Curves were fit using four parameters nonlinear regression. **B** Neutralizing titers of 32 human sera determined by HCIS. Data are shown as mean ± SEM (n = 2). Curves were fit using four parameters nonlinear regression. **C** Half maximal neutralizing dilution fold (ND_50_) of 32 human sera determined by FCM and HCIS. Horizontal lines represent mean ± SEM. **D** Correlation of half maximal neutralizing dilution fold (ND_50_) obtained by FCM and HCIS

Neutralizing antibodies can be detected in the serum of healthy EBV carriers where they persist for a long time. In the case of infectious mononucleosis patients, neutralizing antibodies were detected during the first three weeks and persisted for at least two years [45]. Here we assessed whether the HCIS-based assay could be applied to determine neutralizing titers of healthy EBV carriers’ sera. Sera from 32 healthy carriers were serially diluted and incubated with CNE2-EBV-GFP for 2 h before being incubated with Akata cells for 48 h. Neutralizing titers were determined by the reduction of GFP-positive cells for FCM and the reduction of GFP-positive spots for HCIS. Curves were fit using four parameters nonlinear regression to determine ND_50_ values (Fig. 4A and B). ND_50_ of sera from healthy EBV carriers ranged from 1.5 to 4.5 (Log10) when determined either by FCM or HCIS (Fig. 4C). Remarkably, the neutralizing titers obtained by these two assays were very strongly correlated over a broad range of neutralization efficacy of different sera (R^2^ = 0.8942; Fig. 4D).

To validate the use of the HCIS-based assay to a different gamma-herpesvirus homologous to EBV, we assessed neutralizing titers of sera from rhesus macaques infected by rhesus lymphocryptovirus (rhLCV) against EBV [39]. rhLCV only infects rhesus macaques and the infectious features resemble those of EBV in humans [46]. Twelve rhesus macaque sera showed potent B cell neutralizing activity, as defined by both FCM and HCIS assays against CNE2-EBV-GFP (Additional file 1: Fig. S2A and B). ND_50_ values ranged from 1.5 to 4 (Log10) in both assays (Additional file 1: Fig. S2C). The neutralizing titers measured by HCIS and FCM assays are also very highly correlated (R^2^ = 0.9247; Additional file 1: Fig. S2D). Thus, HCIS is a valid approach to evaluate rhLCV infection in macaques and screen rhLCV-seronegative monkeys for EBV vaccine studies, since the strong cross-reactivity of antibodies targeting glycoproteins of rhLCV can influence EBV vaccine assessment. HCIS may also be used as a high-throughput assay to study cross-reacting rhLCV monoclonal antibodies. Most importantly, HCIS provides a high-throughput method to determine anti-EBV neutralizing titers in sera from humans and rhLCV-infected monkeys.

### Correlation of sera B cell neutralizing titers and anti-gp350 IgG titers

**Fig. 5:**
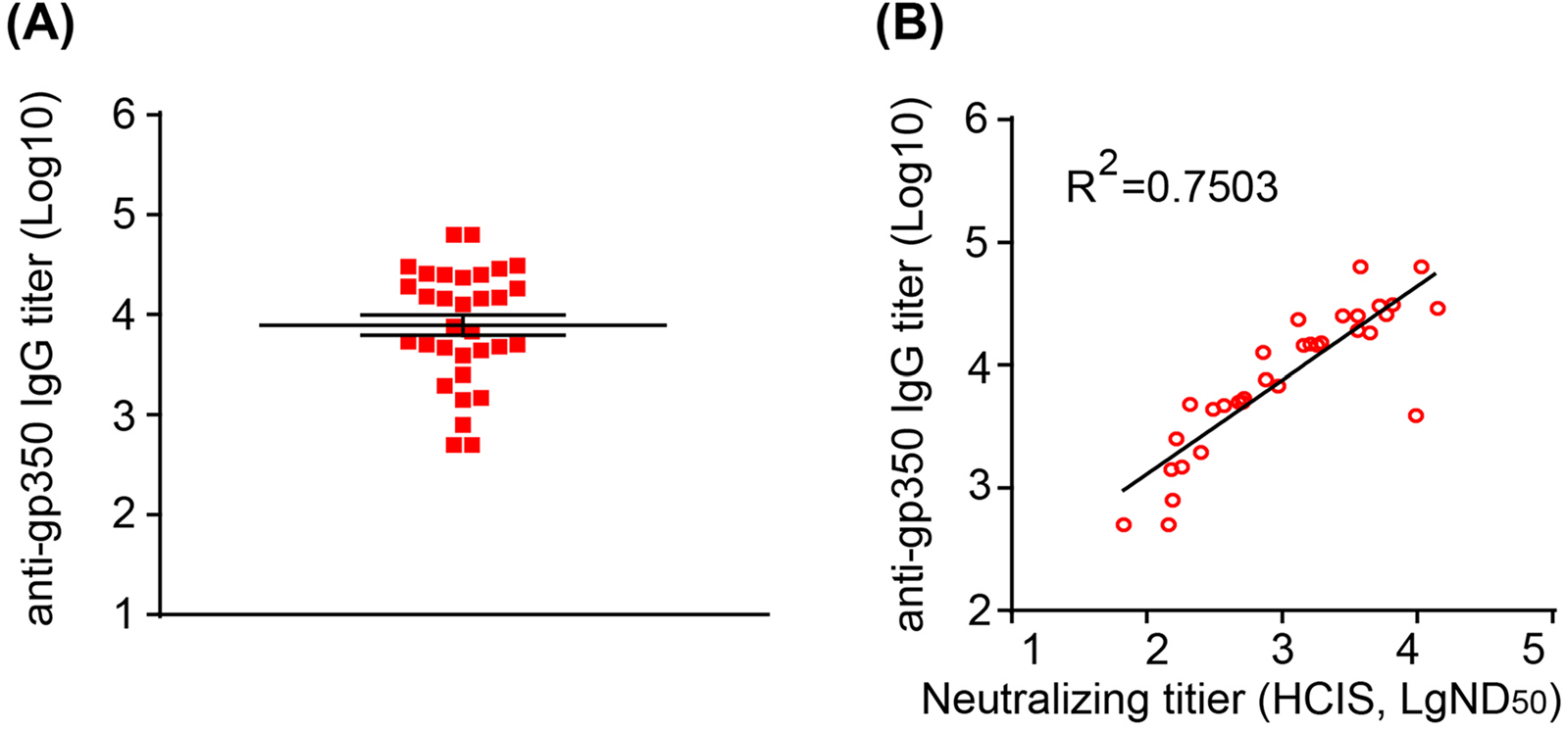
Anti-gp350 IgG titers in human sera and its correlation with B cell neutralizing titer. **A** Anti-gp350 IgG titers in 32 human sera, determined by ELISA. Data are shown as mean ± SEM. **B** Correlation between anti-gp350 IgG titers and B cell neutralizing titers determined by HCIS (Fig. 4C) in human sera

EBV gp350 plays a key role in B cell infection and neutralizing antibodies against gp350 are the major component of B cell neutralization [17, 47]. gp350 is also the most abundant glycoprotein on the virion surface. For these reasons, gp350 is considered an ideal antigen for EBV vaccine development. To define whether B cell neutralizing titers obtained by HCIS and anti-gp350 IgG levels were correlated, we also used ELISA to quantify anti-gp350 IgG titers in the same sera from healthy EBV carriers (Fig. 5A) and rhLCV-infected monkeys (Additional file 1: Fig. S3A). Anti-gp350 titers ranged from 10^2^ to 10^5^, in sera from healthy EBV carriers, and from 10^1.5^ to 10^4^, in monkey sera. The detection of anti-gp350 IgG in rhesus macaques infected by rhLCV is consistent with the cross-reactivity between EBV and rhLCV [48]. Although the correlation is less noticeable at low titers, neutralizing titers determined by HCIS correlated strongly with anti-gp350 IgG titers in sera from healthy EBV carriers or rhLCV-infected monkeys (Figs. 4C and 5B, Additional file 1: Figs. S2C and S3B). These data on human and monkey sera show that the HCIS neutralization assay accurately reflects the importance of anti-gp350 antibodies in neutralizing EBV infection of B cells. Overall, HCIS assays will provide a solid basis to relate the neutralization ability of polyclonal sera with the presence of antigen-specific antibodies, for instance during vaccine trials.

### Production of Akata-EBV-GFP virus

**Fig. 6:**
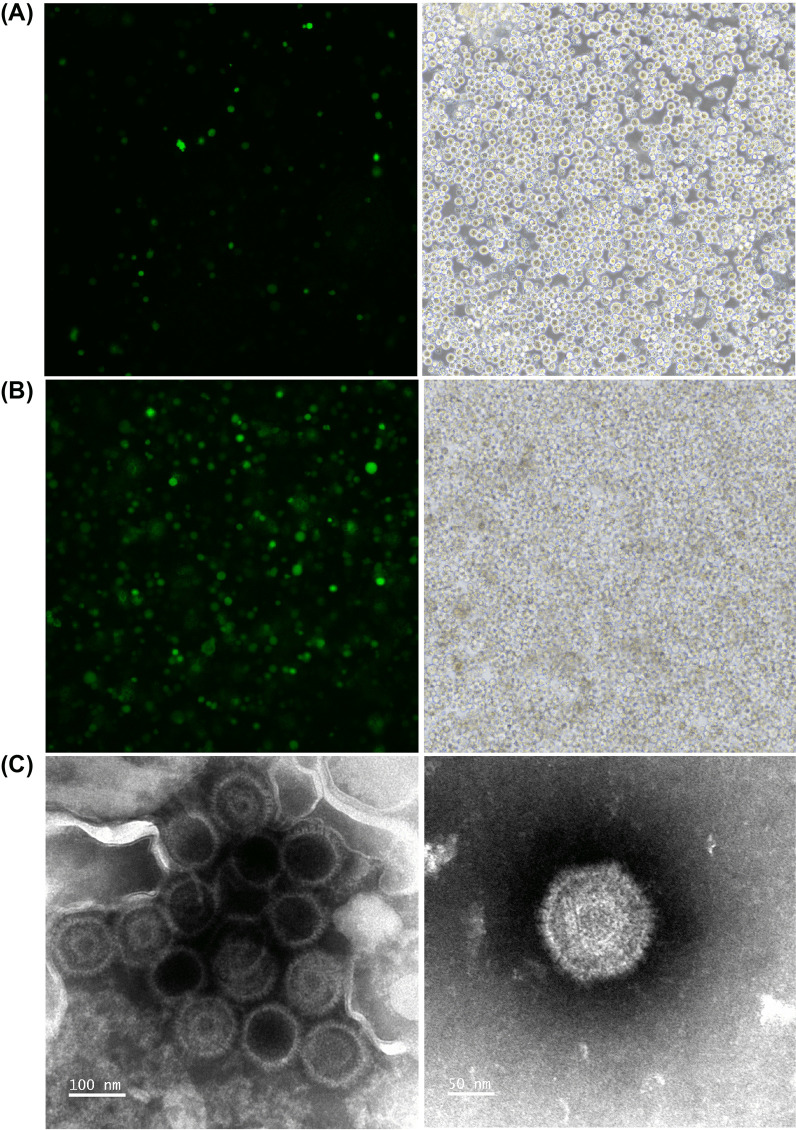
Akata-EBV-GFP virus production and TEM detection. **A** Images of Akata-EBV cells carrying Akata-EBV-GFP before induction with goat anti-human IgG. Few GFP spots were observed indicating that most viruses were in a latent state. **B** Images of Akata-EBV cells carrying Akata-EBV-GFP 72 h after induction. GFP spots were readily observed in most cells indicating viruses were induced to lytic state. (**A**, **B**). Images of cells with the same field of view captured at FITC channel (left panel) and bright-field channel (right panel). **C** TEM images of Akata-EBV-GFP virus. Capsids are visible, but viral envelope did not resist sample processing for TEM. Capsids are visible, but the viral envelope is not observed because sample processing for TEM observation disrupted this membrane structure. The magnification of the left channel is 150,000 x (scale bar: 100 nm) and that of the right channel is 250,000 x (scale bar: 50 nm)

Considering the dual tropism of EBV, it is important to show that the HCIS-based assay is equally valid to determine neutralization titers in epithelial cells. To address that question, we produced the Akata-EBV-GFP virus from Akata-EBV cells. This B cell line carrying the Akata-EBV-GFP genome, can be induced to produce virions, which prefer to infect epithelial cells. Upon induction with goat anti-human IgG, GFP expression increased in Akata-EBV cells after 72 h induction compared with untreated cells (Fig. 6A and B). To confirm the production of the virus in induced cells, concentrated virions were observed by TEM (Fig. 6C).

### Epithelial cell neutralizing titers evaluated by FCM and HCIS

**Fig. 7:**
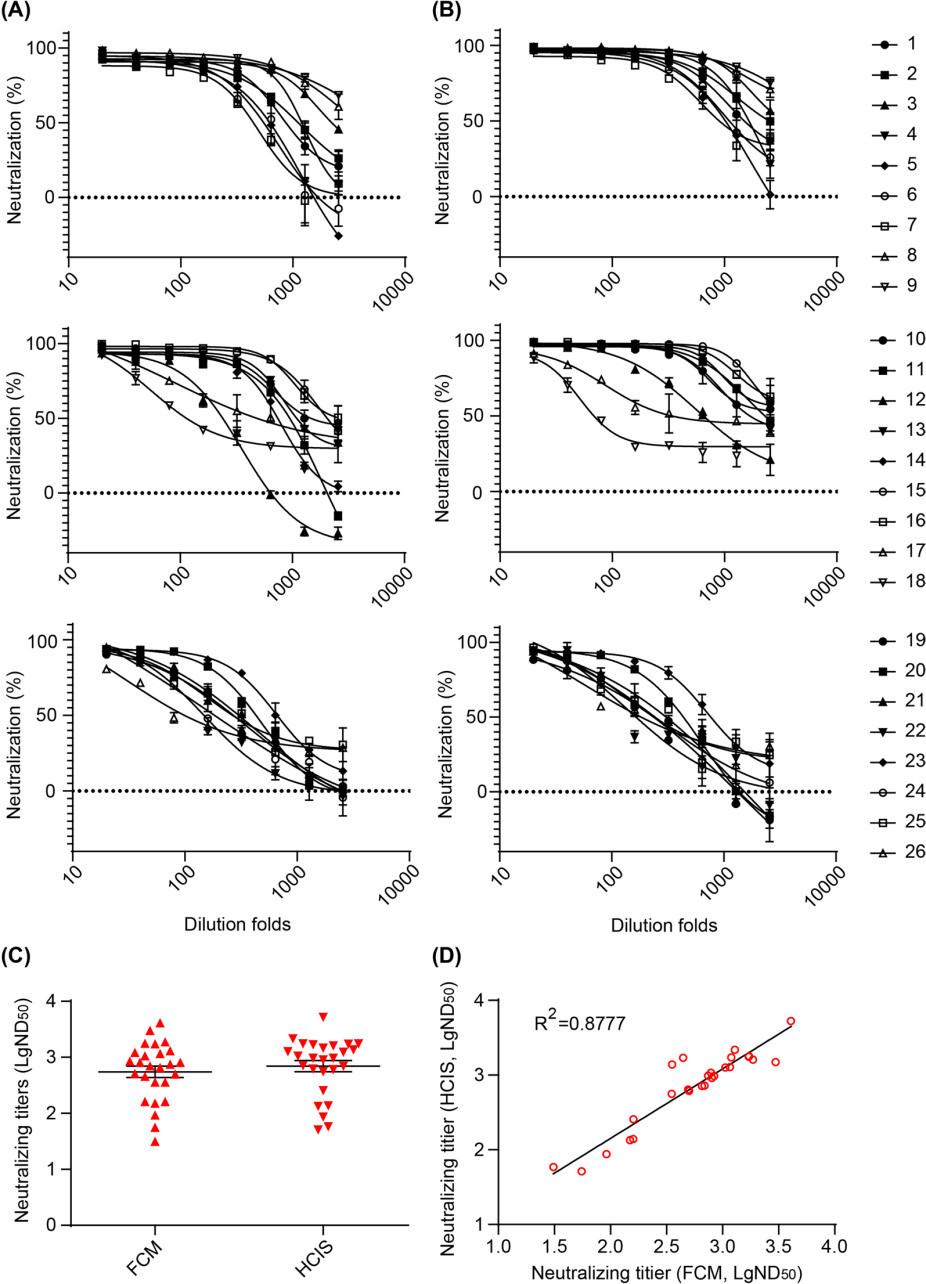
Neutralizing titers of 26 human sera against Akata-EBV-GFP virus infection of epithelial cells determined by FCM and HCIS. **A** Neutralizing titers of 26 human sera determined by FCM. Data was shown as mean ± SEM (n = 2). Curves were fit using four parameters nonlinear regression. **B** Neutralizing titers of 26 human sera determined by HCIS. Data was shown as mean ± SEM (n = 2). Curves were fit using four parameters nonlinear regression. **C** Half maximal neutralizing dilution fold (ND_50_) of 26 human sera determined by FCM and HCIS. Horizontal lines represent mean ± SEM. **D** Correlation of half maximal neutralizing dilution fold (ND_50_) defined between FCM and HCIS

The Akata-EBV-GFP virus was used to assess whether HCIS can be applied to quantify neutralizing titers in epithelial cells. We used the Akata-EBV-GFP virus to infect the EBV-negative epithelial cell line HNE1. Twenty-six serially diluted sera from healthy EBV carriers were incubated with Akata-EBV-GFP virus for 2 h before addition to HNE1 cells for 3 h. After 48 h in culture, neutralizing titers were assessed by FCM and HCIS assays (Fig. 7A, B and Additional file 1: Fig. S4). Neutralization curves were fit using four parameters nonlinear regression (Fig. 7A, B). ND_50_ values obtained from sera of healthy EBV carriers covered a broad range from 10^1^ to 10^4^, detected by FCM and HCIS (Fig. 7C). The neutralizing titers measured by these two assays were remarkably correlated over a broad range of activity (R^2^ = 0.8777; Fig. 7D).

The role of gHgL in EBV entry into epithelial cells is critical. CL59 is a neutralizing monoclonal antibody targeting gHgL, which is known to effectively neutralize epithelial cell infection [27]. CL59 neutralizing titers (NC_50_) determined by FCM and HCIS were similar (Additional file 1: Fig. S5). Overall these data indicate that the HCIS-based assay is a reliable method to quantify the neutralization of monoclonal antibodies for EBV infection of epithelial cells.

### Correlation of epithelial cell neutralizing titers and anti-gHgL IgG titers in human sera

**Fig. 8:**
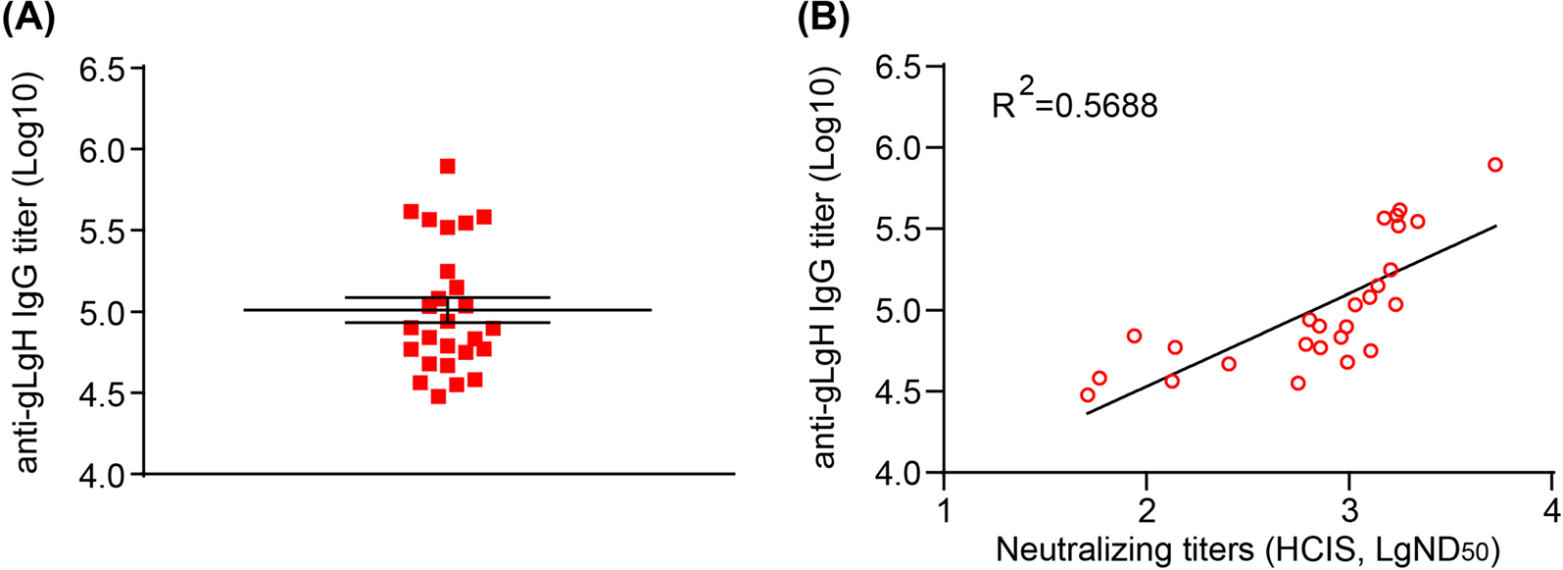
Anti-gHgL IgG titers in human sera and its correlation with epithelial cell neutralizing titer. **A** Anti-gHgL IgG titers in 26 human sera, determined by ELISA. Data are shown as mean ± SEM. **B** Correlation between anti-gHgL IgG titers and epithelial cell neutralizing titers determined by HCIS in human sera

gHgL-specific neutralizing antibodies are the major contributors to the neutralization of EBV infection of epithelial cells [17]. We used ELISA to measure the anti-gHgL IgG titers in sera from healthy EBV carriers (Fig. 8A). Anti-gHgL titers in these healthy EBV carriers correlated positively with neutralizing titers determined by HCIS-based assays (Fig. 7C and 8B).

## Discussion

Here we present a sensitive, high-throughput and robust HCIS-based approach to determine EBV infection titers, as well as neutralizing titers of sera or monoclonal antibodies against infection of B cells and epithelial cells. We validated this new HCIS-based assay by comparing its output to that of an established FCM-based assay [35]. We observed consistent and constant agreement between the two assays in the determination of viral titers, monoclonal antibody neutralizing titers and sera neutralizing titers. We used HCIS to illustrate the significant correlation between gp350 IgG titers and B cell neutralizing titers across multiple sera from healthy EBV carriers and rhLCV-infected monkeys. Likewise, anti-gHgL IgG titers were correlated strongly with epithelial cell neutralizing titers in sera from multiple healthy EBV carriers. This HCIS-based assay can be applied more easily than FCM to high-throughput settings. This assay will be particularly efficient (i) to determine neutralizing titers of large-scale sera samples after vaccine inoculation in pre-clinical and clinical studies, (ii) to screen monoclonal antibodies and characterize their specific neutralization activity, and (iii) to assess the efficacy of EBV-specific antivirals to block B cell or epithelial cell infections. In addition, this HCIS-based assay will facilitate serological and epidemiological studies for large-scale samples to investigate the correlation between neutralizing titers and diseases outcome.

Previously reported methods to determine EBV-specific neutralizing titers include B cell transformation inhibition [32], immunofluorescent-based assay [33], competitive ELISA [34], FCM-based neutralization assay [35] and FIA-based neutralization assay [36, 37]. Comparatively, FIA-based assays are more amenable to high-throughput settings. However, the published FIA assay relied on a CR2 overexpressing epithelial cell line to mimic B cell infection by EBV. Considering the dual tropism of EBV, it is necessary to consider a high-throughput approach applicable to B cells as well as epithelial cells. The HCIS-based assay described here has been validated in those two settings. First, the EBV-negative B cell line Akata was used in combination with the epithelial cell-derived CNE2-EBV-GFP virus. Second, the EBV negative epithelial cell line, HNE1 was used in combination with the B cell-derived Akata-EBV-GFP virus. These two models more realistically simulate the process of EBV natural infection. Neutralizing titers determined under these conditions are therefore more reliable. This assay also allowed accurate testing neutralization of antibodies against glycoproteins involved in infection of B cells (i.e. gp350) or epithelial cells (i.e. gHgL). However, the premise of an accurate analysis of GFP positive cells with HCIS is that the cells are single dispersed and evenly distributed in the wells. Therefore, epithelial cells need to be cultured in 96 well plates at a lower density for this assay. For B cells infection model, cells are clustered around the edges because of the edge effect of 96 well plates. It is necessary to shake the plate to disperse the cells and let them be evenly distributed in the well before imaging. Otherwise, the results will not be accurate.

Antibodies targeting different EBV proteins are raised with different peak times after infection [45]. Importantly, high levels of neutralizing titers and high anti-gp350 IgG titers are considered low risk biomarkers for the development of NPC [49]. It is known that gp350-specific neutralizing antibodies are the major contributors to B cell neutralization in healthy individuals [17, 47]. Indeed, in this study B cell neutralizing titers of healthy EBV carriers determined by HCIS correlated strongly with anti-gp350 IgG titers determined by ELISA. A similar positive correlation was observed in sera from monkeys infected with rhLCV. Although no gp350-based vaccine has been approved yet, gp350 remains a major candidate for vaccine development when combined with a more efficient adjuvant such as AS01_B_, Matrix-M and 3 M-052 [50–52]. As for epithelial cell infection, gHgL specific neutralizing antibodies contributed to ~ 75% of the neutralizing activity [17]. Five monoclonal antibodies targeting gHgL have been reported, which are AMMO1 (human) [24], 6H2 (mouse) [25], 1D8 (human) [26], CL40 (mouse) [27] and CL59 (mouse) [27]. AMMO1 binds to gH domain I and II, 6H2 binds to gH domain IV and 1D8 binds to gH domain II. All three antibodies potently neutralize both B cell and epithelial cell infection. On the other hand, CL40 (domain II) and CL59 (domain I) only efficiently block epithelial cell infection. Interestingly, AMMO1, 1D8 and 6H2 antibodies protected humanized mice against EBV infection while 72A1 (against gp350) failed to reduce viral load in vivo [25, 26, 53]. Here, using HCIS, we also demonstrated a strong correlation between anti-gHgL IgG titers and epithelial cell neutralizing titers in sera from multiple healthy EBV carriers. The gHgL complex participates in the infection process of epithelial and B cells as an activator of the membrane fusion effector gB. Consequently, gHgL needs to be taken into account for vaccine design. Indeed, antibodies induced by gHgL-ferritin nanoparticles were highly efficient at neutralizing infection of epithelial cells [17]. gHgL-ferritin nanoparticles induced neutralizing antibodies in BALB/c mice and cynomolgus macaques and antibodies purified from immunized mice passively protected humanized mice from lethal EBV challenge [17, 54].

## Conclusions

A lot of work remains to characterize serological profiles in EBV infected individuals and to develop prophylactic or therapeutic agents against EBV. Therefore, a high-throughput method to quantify EBV neutralization is urgently needed to facilitate studies of EBV infection in the human population. The high-throughput HCIS-based assay reported here has been designed and tested to address this need. It will be an asset in the development of prophylactic and therapeutic agents against EBV infection, and it will facilitate serological and epidemiological investigations of large-scale samples to study the relationship between neutralizing antibodies and disease risks.

## Supplementary Information

**Additional file 1**.** Figure S1**. CNE2-EBV-GFP infection of Akata cells was determined by FCM and HCIS.** Figure S2**. Neutralizing titers of 12 rhesus macaque sera against CNE2-EBV-GFP virus infection of B cells determined by FCM and HCIS.** Figure S3**. Anti-gp350 IgG titer in rhesus macaque monkey sera and its correlation with B cell neutralizing titer.** Figure S4**. Akata-EBV-GFP infection of HNE1 cells was determined by FCM and HCIS.** Figure S5**. Neutralizing activity of CL59 determined by FCM and HCIS.

## Data Availability

The data that support the findings of this study are available from the corresponding author upon reasonable request.

## References

[1] Young LS, Yap LF, Murray PG (2016). Epstein-Barr virus: more than 50 years old and still providing surprises. Nat Rev Cancer.

[2] Houen G, Trier NH (2020). Epstein-Barr virus and systemic autoimmune diseases. Front Immunol.

[3] Bjornevik K, Cortese M, Healy BC, Kuhle J, Mina MJ, Leng Y, Elledge SJ, Niebuhr DW, Scher AI, Munger KL, Ascherio A (2022). Longitudinal analysis reveals high prevalence of Epstein-Barr virus associated with multiple sclerosis. Science.

[4] Khan G, Fitzmaurice C, Naghavi M, Ahmed LA (2020). Global and regional incidence, mortality and disability-adjusted life-years for Epstein-Barr virus-attributable malignancies, 1990–2017. BMJ Open.

[5] Borza CM, Hutt-Fletcher LM (2002). Alternate replication in B cells and epithelial cells switches tropism of Epstein-Barr virus. Nat Med.

[6] Tanner J, Weis J, Fearon D, Whang Y, Kieff E (1987). Epstein-Barr virus gp350/220 binding to the B lymphocyte C3d receptor mediates adsorption, capping, and endocytosis. Cell.

[7] Ogembo JG, Kannan L, Ghiran I, Nicholson-Weller A, Finberg RW, Tsokos GC, Fingeroth JD (2013). Human complement receptor type 1/CD35 is an Epstein-Barr virus receptor. Cell Rep.

[8] Mohl BS, Chen J, Longnecker R: Gammaherpesvirus entry and fusion: A tale how two human pathogenic viruses enter their host cells. Adv Virus Res 2019;104:313–43. 10.1016/bs.aivir.2019.05.006 31439152

[9] Tugizov SM, Berline JW, Palefsky JM (2003). Epstein-Barr virus infection of polarized tongue and nasopharyngeal epithelial cells. Nat Med.

[10] Zhang H, Li Y, Wang HB, Zhang A, Chen ML, Fang ZX, Dong XD, Li SB, Du Y, Xiong D (2018). Ephrin receptor A2 is an epithelial cell receptor for Epstein-Barr virus entry. Nat Microbiol.

[11] Zhang H, Li Y, Wang HB, Zhang A, Chen ML, Fang ZX, Dong XD, Li SB, Du Y, Xiong D (2018). Author Correction: Ephrin receptor A2 is an epithelial cell receptor for Epstein-Barr virus entry. Nat Microbiol.

[12] De Paschale M, Clerici P (2012). Serological diagnosis of Epstein-Barr virus infection: Problems and solutions. World J Virol.

[13] Henle W, Henle G, Andersson J, Ernberg I, Klein G, Horwitz CA, Marklund G, Rymo L, Wellinder C, Straus SE (1987). Antibody responses to Epstein-Barr virus-determined nuclear antigen (EBNA)-1 and EBNA-2 in acute and chronic Epstein-Barr virus infection. Proc Natl Acad Sci U S A.

[14] Coghill AE, Hildesheim A (2014). Epstein-Barr virus antibodies and the risk of associated malignancies: review of the literature. Am J Epidemiol.

[15] Huang Y, Rao H, Yan S, Wang F, Wu Q, Feng Y, Zhang Y (2017). Serum EBV EA-IgA and VCA-IgA antibodies can be used for risk group stratification and prognostic prediction in extranodal NK/T cell lymphoma: 24-year experience at a single institution. Ann Hematol.

[16] Zhu QY, Kong XW, Sun C, Xie SH, Hildesheim A, Cao SM, Zeng MS (2020). Association between antibody responses to Epstein-Barr virus glycoproteins, neutralization of infectivity, and the risk of nasopharyngeal carcinoma. mSphere.

[17] Bu W, Joyce MG, Nguyen H, Banh DV, Aguilar F, Tariq Z, Yap ML, Tsujimura Y, Gillespie RA, Tsybovsky Y (2019). Immunization with components of the viral fusion apparatus elicits antibodies that neutralize epstein-barr virus in B cells and epithelial cells. Immunity.

[18] Sun C, Chen XC, Kang YF, Zeng MS (2021). The Status and Prospects of Epstein-Barr Virus Prophylactic Vaccine Development. Front Immunol.

[19] Gu SY, Huang TM, Ruan L, Miao YH, Lu H, Chu CM, Motz M, Wolf H (1995). First EBV vaccine trial in humans using recombinant vaccinia virus expressing the major membrane antigen. Dev Biol Stand.

[20] Moutschen M, Leonard P, Sokal EM, Smets F, Haumont M, Mazzu P, Bollen A, Denamur F, Peeters P, Dubin G, Denis M (2007). Phase I/II studies to evaluate safety and immunogenicity of a recombinant gp350 Epstein-Barr virus vaccine in healthy adults. Vaccine.

[21] Sokal EM, Hoppenbrouwers K, Vandermeulen C, Moutschen M, Leonard P, Moreels A, Haumont M, Bollen A, Smets F, Denis M (2007). Recombinant gp350 vaccine for infectious mononucleosis: a phase 2, randomized, double-blind, placebo-controlled trial to evaluate the safety, immunogenicity, and efficacy of an Epstein-Barr virus vaccine in healthy young adults. J Infect Dis.

[22] Rees L, Tizard EJ, Morgan AJ, Cubitt WD, Finerty S, Oyewole-Eletu TA, Owen K, Royed C, Stevens SJ, Shroff RC (2009). A phase I trial of epstein-barr virus gp350 vaccine for children with chronic kidney disease awaiting transplantation. Transplantation.

[23] Hoffman GJ, Lazarowitz SG, Hayward SD (1980). Monoclonal antibody against a 250,000-dalton glycoprotein of Epstein-Barr virus identifies a membrane antigen and a neutralizing antigen. Proc Natl Acad Sci U S A.

[24] Snijder J, Ortego MS, Weidle C, Stuart AB, Gray MD, McElrath MJ, Pancera M, Veesler D, McGuire AT (2018). An antibody targeting the fusion machinery neutralizes dual-tropic infection and defines a site of vulnerability on Epstein-Barr virus. Immunity.

[25] Hong J, Zhong L, Zheng Q, Wu Q, Zha Z, Wei D, Chen H, Zhang W, Zhang S, Huang Y (2022). A neutralizing antibody targeting gH provides potent protection against EBV challenge in vivo. J Virol.

[26] Zhu QY, Shan S, Yu J, Peng SY, Sun C, Zuo Y, Zhong LY, Yan SM, Zhang X, Yang Z (2021). A potent and protective human neutralizing antibody targeting a novel vulnerable site of Epstein-Barr virus. Nat Commun.

[27] Sathiyamoorthy K, Jiang J, Mohl BS, Chen J, Zhou ZH, Longnecker R, Jardetzky TS (2017). Inhibition of EBV-mediated membrane fusion by anti-gHgL antibodies. Proc Natl Acad Sci U S A.

[28] Sathiyamoorthy K, Hu YX, Mohl BS, Chen J, Longnecker R, Jardetzky TS (2016). Structural basis for Epstein-Barr virus host cell tropism mediated by gp42 and gHgL entry glycoproteins. Nat Commun.

[29] Strnad BC, Schuster T, Klein R, Hopkins RF, Witmer T, Neubauer RH, Rabin H (1982). Production and characterization of monoclonal antibodies against the Epstein-Barr virus membrane antigen. J Virol.

[30] Zhang X, Hong J, Zhong L, Wu Q, Zhang S, Zhu Q, Chen H, Wei D, Li R, Zhang W (2022). Protective anti-gB neutralizing antibodies targeting two vulnerable sites for EBV-cell membrane fusion. Proc Natl Acad Sci U S A.

[31] Hong J, Wei D, Zhong L, Wu Q, Chen K, Zhang W, Yang Y, Chen J, Xia N, Zhang X, Chen Y (2022). Glycoprotein B antibodies completely neutralize EBV infection of B Cells. Front Immunol.

[32] Miller G, Niederman JC, Stitt DA (1972). Infectious mononucleosis: appearance of neutralizing antibody to Epstein-Barr virus measured by inhibition of formation of lymphoblastoid cell lines. J Infect Dis.

[33] Rocchi G, Hewetson JF (1973). A practical and quantitative microtest for determination of neutralizing antibodies against Epstein-Barr virus. J Gen Virol.

[34] Wilson AD, Morgan AJ (1998). Indirect measurement of Epstein-Barr virus neutralising antibodies by ELISA. J Virol Methods.

[35] Sashihara J, Burbelo PD, Savoldo B, Pierson TC, Cohen JI (2009). Human antibody titers to Epstein-Barr Virus (EBV) gp350 correlate with neutralization of infectivity better than antibody titers to EBV gp42 using a rapid flow cytometry-based EBV neutralization assay. Virology.

[36] Lin R, Heeke D, Liu H, Rao E, Marshall JD, Chio V, Cataniag F, Yu L, Zuo F, McCarthy MP (2017). Development of a robust, higher throughput green fluorescent protein (GFP)-based Epstein-Barr Virus (EBV) micro-neutralization assay. J Virol Methods.

[37] Liu H, Gemmell L, Lin R, Zuo F, Balfour HH, Woo JC, Hayes GM (2020). Development of an improved Epstein-Barr virus (EBV) neutralizing antibody assay to facilitate development of a prophylactic gp350-subunit EBV vaccine. Mediterr J Hematol Infect Dis.

[38] Giuliano KA, Haskins JR, Taylor DL (2003). Advances in high content screening for drug discovery. Assay Drug Dev Technol.

[39] Rivailler P, Jiang H, Cho YG, Quink C, Wang F (2002). Complete nucleotide sequence of the rhesus lymphocryptovirus: genetic validation for an Epstein-Barr virus animal model. J Virol.

[40] Carville A, Mansfield KG (2008). Comparative pathobiology of macaque lymphocryptoviruses. Comp Med.

[41] Zhan F, Jiang N, Cao L, Deng L, Tan G, Zhou M, Xie Y, Li G (1998). Primary study of differentially expressed cDNA sequences in cell line HNE1 of human nasopharyngeal carcinoma by cDNA representational difference analysis. Zhonghua Yi Xue Yi Chuan Xue Za Zhi.

[42] Zhang HJ, Tian J, Qi XK, Xiang T, He GP, Zhang H, Yu X, Zhang X, Zhao B, Feng QS (2018). Epstein-Barr virus activates F-box protein FBXO2 to limit viral infectivity by targeting glycoprotein B for degradation. PLoS Pathog.

[43] Molesworth SJ, Lake CM, Borza CM, Turk SM, Hutt-Fletcher LM (2000). Epstein-Barr virus gH is essential for penetration of B cells but also plays a role in attachment of virus to epithelial cells. J Virol.

[44] Szakonyi G, Klein MG, Hannan JP, Young KA, Ma RZ, Asokan R, Holers VM, Chen XS (2006). Structure of the Epstein-Barr virus major envelope glycoprotein. Nat Struct Mol Biol.

[45] Bu W, Hayes GM, Liu H, Gemmell L, Schmeling DO, Radecki P, Aguilar F, Burbelo PD, Woo J, Balfour HH, Cohen JI (2016). Kinetics of Epstein-Barr virus (EBV) neutralizing and virus-specific antibodies after primary infection with EBV. Clin Vaccine Immunol.

[46] Moghaddam A, Rosenzweig M, Lee-Parritz D, Annis B, Johnson RP, Wang F (1997). An animal model for acute and persistent Epstein-Barr virus infection. Science.

[47] Thorley-Lawson DA, Poodry CA (1982). Identification and isolation of the main component (gp350-gp220) of Epstein-Barr virus responsible for generating neutralizing antibodies in vivo. J Virol.

[48] Muhe J, Aye PP, Quink C, Eng JY, Engelman K, Reimann KA, Wang F (2021). Neutralizing antibodies against Epstein-Barr virus infection of B cells can protect from oral viral challenge in the rhesus macaque animal model. Cell Rep Med.

[49] Coghill AE, Bu W, Nguyen H, Hsu WL, Yu KJ, Lou PJ, Wang CP, Chen CJ, Hildesheim A, Cohen JI (2016). High levels of antibody that neutralize B-cell infection of Epstein-Barr Virus and that bind EBV gp350 are associated with a lower risk of nasopharyngeal carcinoma. Clin Cancer Res.

[50] Didierlaurent AM, Laupeze B, Di Pasquale A, Hergli N, Collignon C, Garcon N (2017). Adjuvant system AS01: helping to overcome the challenges of modern vaccines. Expert Rev Vaccines.

[51] Keech C, Albert G, Cho I, Robertson A, Reed P, Neal S, Plested JS, Zhu M, Cloney-Clark S, Zhou H (2020). Phase 1–2 trial of a SARS-CoV-2 recombinant spike protein nanoparticle vaccine. N Engl J Med.

[52] Kasturi SP, Rasheed MAU, Havenar-Daughton C, Pham M, Legere T, Sher ZJ, Kovalenkov Y, Gumber S, Huang JY, Gottardo R (2020). 3M–052, a synthetic TLR-7/8 agonist, induces durable HIV-1 envelope-specific plasma cells and humoral immunity in nonhuman primates. Sci Immunol.

[53] Singh S, Homad LJ, Akins NR, Stoffers CM, Lackhar S, Malhi H, Wan YH, Rawlings DJ, McGuire AT (2020). Neutralizing antibodies protect against oral transmission of lymphocryptovirus. Cell Rep Med.

[54] Malhi H, Homad LJ, Wan YH, Poudel B, Fiala B, Borst AJ, Wang JY, Walkey C, Price J, Wall A (2022). Immunization with a self-assembling nanoparticle vaccine displaying EBV gH/gL protects humanized mice against lethal viral challenge. Cell Rep Med.

