# Supplementary material for: A high-throughput neutralizing assay for antibodies and sera evaluation against Epstein-Barr virus
Source: Virol J. 2022 Nov 23;19:196. doi: 10.1186/s12985-022-01911-1 (PMC9685953; doi:10.1186/s12985-022-01911-1)
Supplement: Supplementary file 1 — Additional file 1. Figure S1. CNE2-EBV-GFP infection of Akata cells was determined by FCM and HCIS. Figure S2. Neutralizing titers of 12 rhesus macaque sera against CNE2-EBV-GFP virus infection of B cells determined by FCM and HCIS. Figure S3. Anti-gp350 IgG titer in rhesus macaque monkey sera and its correlation with B cell neutralizing titer. Figure S4. Akata-EBV-GFP infection of HNE1 cells was determined by FCM and HCIS. Figure S5. Neutralizing activity of CL59 determined by FCM and HCIS. [file 12985_2022_1911_MOESM1_ESM.docx]

**Supplementary figures**


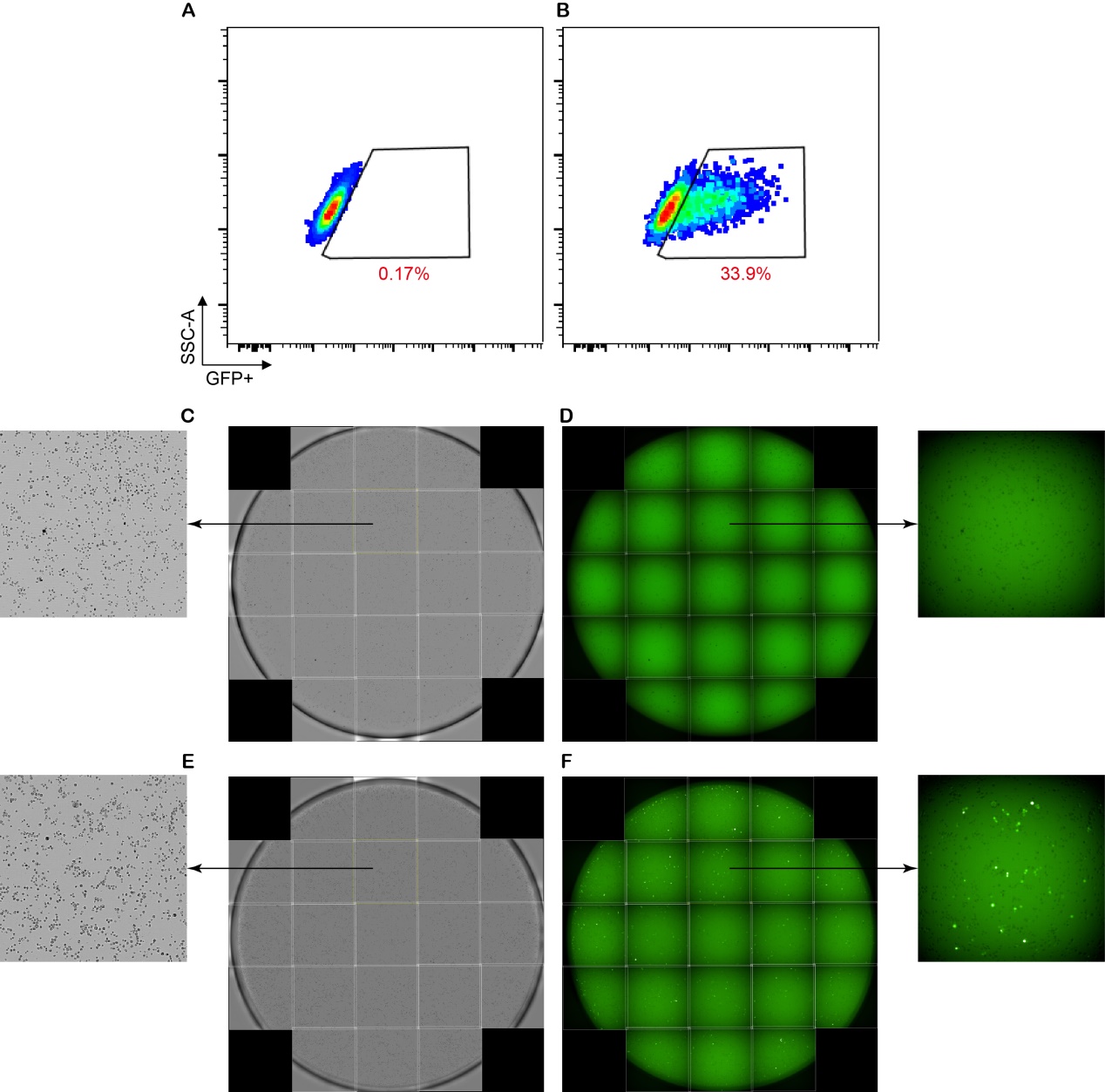


**Figure S1. CNE2-EBV-GFP infection of Akata cells was determined by FCM and HCIS**.

(A) and (B). Representative FCM plots of uninfected Akata cells (negative control) and Akata cells after 48h infection. GFP positive cells representing infected cells were gated according to the negative control.

(C) and (D). Representative HCIS images of bright field (C) and GFP channel (D) for uninfected Akata cells (negative control). Each well was divided to 21 fields of view and images were captured for each field.

(E) and (F). Representative HCIS images of bright field (E) and GFP channel (F) for Akata cells after 48h infection. Each well was divided to 21 fields of view and images were captured for each field. Infected cells were calculated by HCIS programs defined GFP positive spots. Each GFP spot represents a single cell.


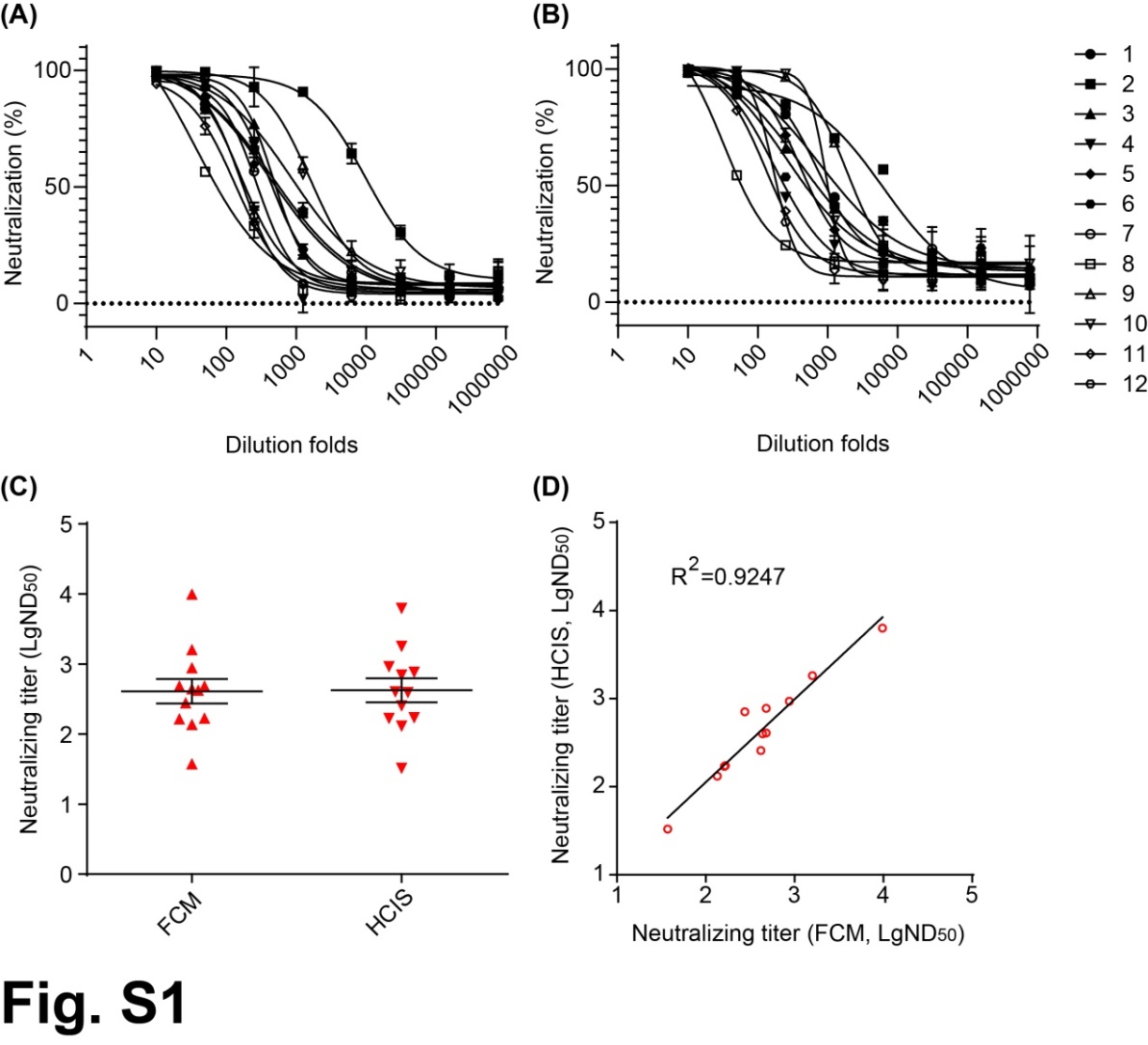


**Figure S2. Neutralizing titers of 12** **rhesus macaque sera against CNE2-EBV-GFP virus infection of B cells determined by FCM and HCIS.**

(A). Neutralizing titers of 12 rhesus macaque sera determined by FCM. Data was shown as mean ± SEM (n=2). Curves were fit using four parameters nonlinear regression.

(B). Neutralizing titers of 12 rhesus macaque sera determined by HCIS. Data was shown as mean ± SEM (n=2). Curves were fit using four parameters nonlinear regression.

(C). Half maximal neutralizing dilution fold (ND_50_) of 12 monkey sera determined by FCM and HCIS, respectively. Data was shown as mean ± SEM.

(D). Correlation of half maximal neutralizing dilution fold (ND_50_) defined between FCM and HCIS.


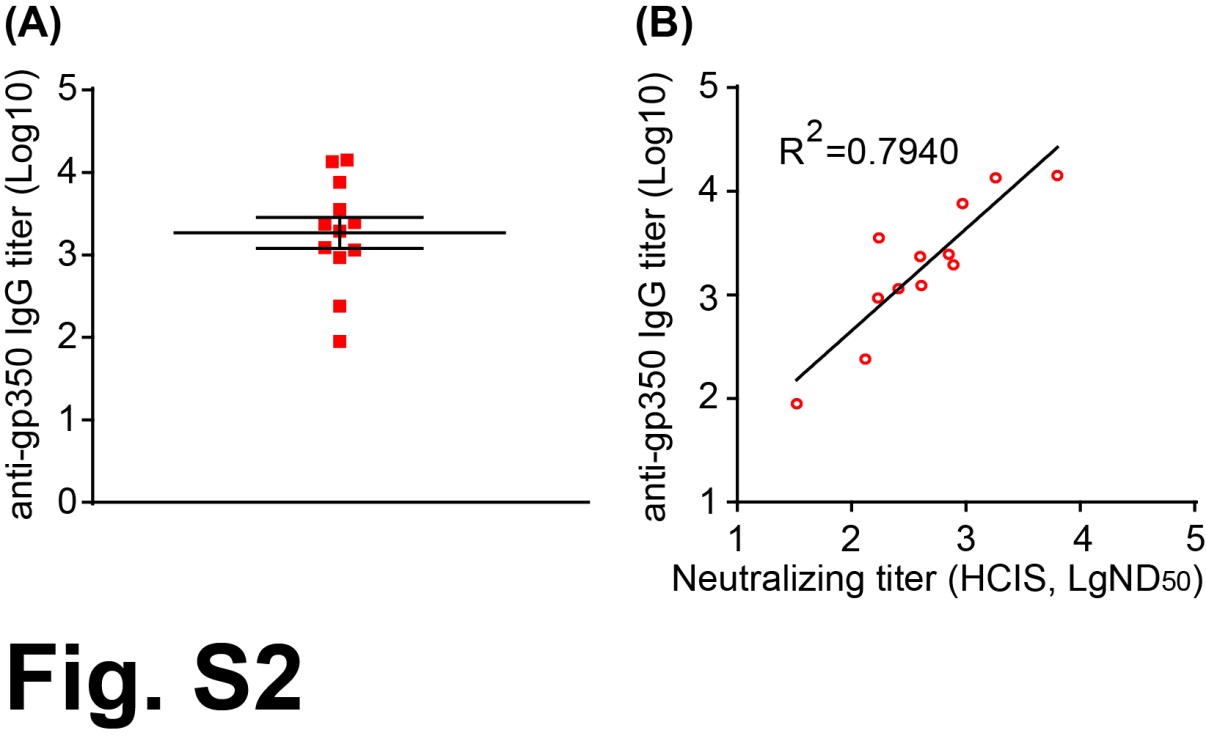


**Figure S3. Anti-gp350 IgG titer in rhesus macaque monkey sera and its correlation with B cell neutralizing titer.**

(A). Anti-gp350 IgG titers of 12 rhesus macaque sera. Data are shown as mean ± SEM.

(B). Correlation between anti-gp350 IgG titers and B cell neutralizing titers determined by HCIS in rhesus macaque sera.


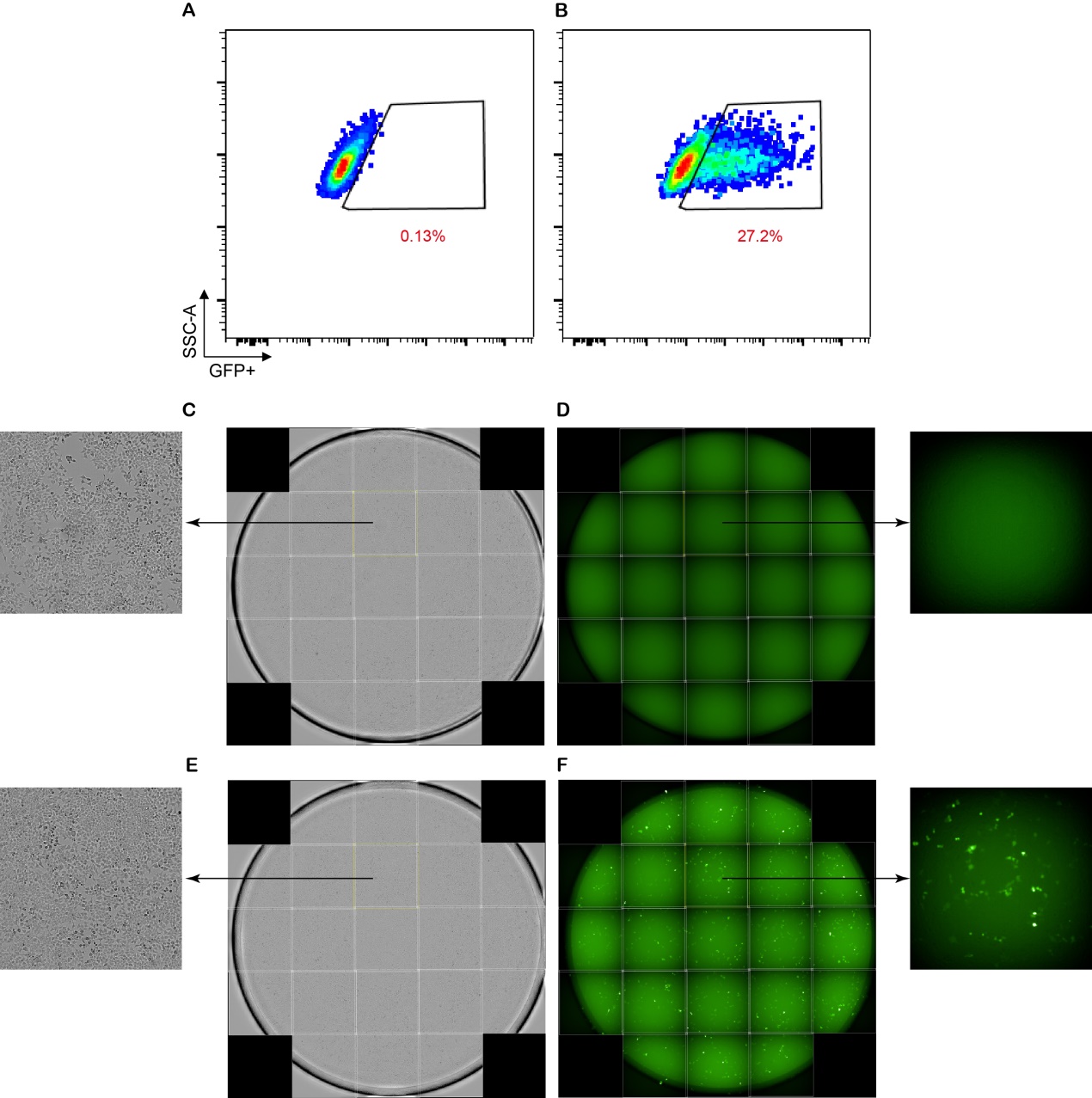


**Figure S4. Akata-EBV-GFP infection of HNE1 cells was determined by FCM and HCIS.**

(A) and (B). Representative FCM plots of uninfected HNE1 cells (negative control) and HNE1 cells after 48h infection. GFP positive cells representing infected cells were gated according to the negative control.

(C) and (D). Representative HCIS images of bright field (C) and GFP channel (D) for uninfected HNE1 cells (negative control). Each well was divided to 21 fields of view and images were captured for each field.

(E) and (F). Representative HCIS images of bright field (E) and GFP channel (F) for HNE1 cells after 48h infection. Each well was divided to 21 fields of view and images were captured for each field. Infected cells were calculated by HCIS programs defined GFP positive spots. Each GFP spot represents a single cell.


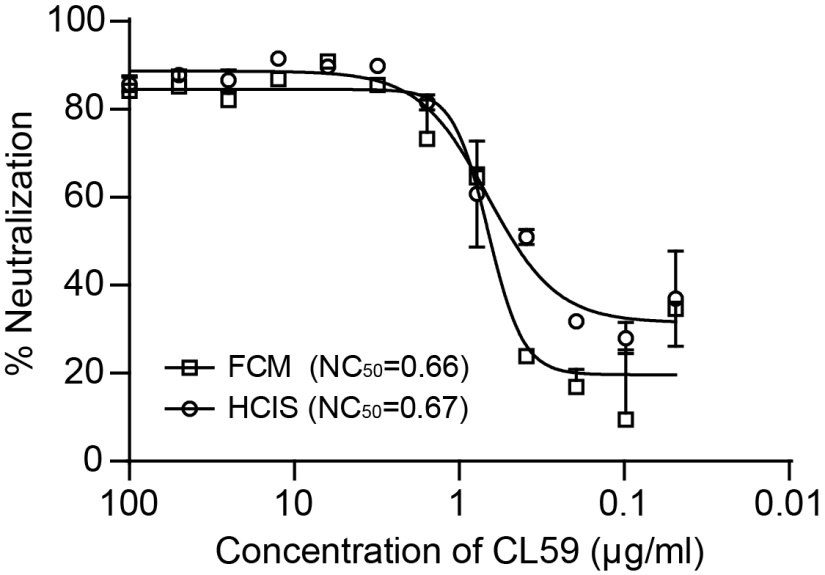


**Figure S5. Neutralizing activity of CL59 determined by FCM and HCIS.** Data was shown as mean ± SEM. Curve was fit using four parameters nonlinear regression. NC_50_: half maximal neutralizing concentration.
